# Cents and shenshibility: The role of reward in talker-specific phonetic recalibration

**DOI:** 10.3758/s13414-025-03048-z

**Published:** 2025-04-11

**Authors:** Hannah Mechtenberg, Shawn N. Cummings, Emily B. Myers, Sahil Luthra

**Affiliations:** 1 Department of Psychological Sciences, University of Connecticut, Storrs, CT USA; 2 Department of Speech, Language and Hearing Sciences, University of Connecticut, Storrs, CT USA; 3 Department of Psychology, Carnegie Mellon University, Pittsburgh, PA USA

## Abstract

To what degree might speech perception be affected by factors external to the speech signal? Here, we consider whether listeners consider the value of individual talkers (e.g., a person that is important to the listener versus a passing stranger) when learning the idiosyncrasies of their speech, perhaps by directing their attention to the more “valuable” talker. In general, talker-specific phonetic recalibration has proven to be remarkably robust, leaving open how permeable it is to domain-general influences like attention and reward. The current study asked whether the magnitude of the talker-specific learning effect was modulated by an external reward intended to bias listeners to prioritize one talker over the other. Using an established paradigm, participants learned idiosyncratic pronunciations of the /s/ and /ʃ/ fricatives by two talkers—“Jane” and “Austin.” Over multiple experiments, participants were more likely to receive a few additional cents for one talker compared with the other following correct responses during exposure. We hypothesized that participants would show greater phonetic recalibration to the high-reward talker compared with the low-reward talker. Our results showed that participants did not show a difference in the degree of learning based on the value of each talker, suggesting that lexically guided perceptual learning is robust to external rewards. This finding opens discussions into the role of reward and attention in talker-specific phonetic recalibration, and how domain-general factors influence speech perception more broadly.

**Supplementary Information:**

The online version contains supplementary material available at 10.3758/s13414-025-03048-z.

## Introduction

As pro-social creatures, humans have a drive to communicate (e.g., Tomasello, 2010). In addition to the primary goal of conveying information, language is also used to solidify social alliances (e.g., Giles et al., 1991) and signal our place in the social hierarchy (e.g., Labov, 1964). As such, it is not surprising that in conversation, listeners are judicious in how they allocate their attentional resources to different interlocutors. At a crowded party, for instance, you might find yourself attempting to eavesdrop on a conversation from a compelling new guest who has just arrived, while barely attending to the bore you have been introduced to. In cocktail party-like settings, listeners can selectively allocate auditory attention toward a talker or speech content that has high value to the listener (e.g., Cherry, 1953; Fitzroy et al., 2020); such attention is directed in a volitional (i.e., top-down) way (e.g., Shinn-Cunningham, 2008). Of interest is how a listener’s motivation, driven by either intrinsic or extrinsic factors, to engage with the speech of a particular talker might modulate *other* aspects of speech perception. In particular, it is tempting to assume that the perceived “value” of a talker might influence not only selective auditory attention but also what a listener learns about the specific phonetic idiosyncrasies of the talker’s voice. In the current study, we test the idea that the perceived value of a talker might shape how listeners direct attention to a talker, with consequences for how listeners learn the idiosyncrasies of that talker’s speech.

The perceived value of a stimulus has been known to drive shifts in selective attention in perception more generally. Value-based attentional capture is thought to arise from an automatic value signal that biases perceivers to attend to reward-linked stimuli, even if those stimuli are orthogonal to current task goals (Anderson et al., 2011). For instance, when participants learn to associate a reward with a particular feature of a stimulus (e.g., the color red) during training, that feature can become highly distracting in later tasks for which it is irrelevant (for review of the phenomenon, see Anderson, 2016a; Anderson & Halpern, 2017). In a study by Anderson et al. (2016c), high and low monetary rewards were incidentally associated with specific stimuli in an auditory detection task. During a subsequent task in which these reward-associated stimuli were not relevant, inclusion of the previously rewarded stimuli drew participant attention away from the current task. Further, the degree of distraction was modulated by the value (high or low) of the rewarded stimuli. This finding suggests that stimuli previously associated with rewards receive continued attentional priority and can dynamically bias selective attention contingent on the magnitude of the associated reward, motivating perceivers to prioritize one signal over another (Bourgeois et al., 2016; P. Zhang et al., 2018). While preliminary evidence points to a complex role of reward during perceptual category learning of novel phonetic categories (Roark & Chandrasekaran, 2024), it has not to our knowledge been tested how reward-mediated biases in selective attention may differentially influence how listeners learn the details of multiple talkers’ speech.

Speech perception is a complex process, made more difficult due to the considerable variability in how individual talkers produce their speech sounds (e.g., Hillenbrand et al., 1995; Peterson & Barney, 1952). Fortunately, listeners are generally quite adept at learning the phonetic idiosyncrasies of a given talker. A long line of work investigating phonetic recalibration to different talkers, and specifically recalibration driven by lexical information, demonstrates this phenomenon (e.g., Eisner & McQueen, 2005, 2006; Kraljic & Samuel, 2007; Norris et al., 2003). For instance, say you are conversing with two people. “Jane” produces her /s/ sounds more like /ʃ/ (e.g., *absent* is closer to *ab****sh****ent*) while “Austin” pronounces his /ʃ/ sounds more like /s/ (e.g., *publisher* approaches *publi****s****er*). The listener can uses their lexical knowledge (e.g., *absent* is a word, but *abshent* is not) to resolve ambiguity in their input (Ganong, 1980). This experience also encourages the listener to adjust their expectations for subsequent productions by the same talker. Experiments in lexically guided perceptual learning often assess these adjustments by later presenting ambiguous segments in a context where lexical knowledge cannot resolve ambiguity. In this example, a listener might demonstrate having learned Jane’s /ʃ/-like /s/ by categorizing an ambiguous stimulus between *sign* or *shine* more often as *sign*. Notably, the vast majority of work in this domain has examined learning as a binary outcome. However, it can be informative to also consider whether the extent of learning may be larger or smaller in some circumstances (Cummings & Theodore, 2023; Luthra et al., 2021a, 2021b).

Listeners conceivably cannot attend to everything in their environment and thus must make (perhaps implicit) choices as to which speech signals may be more valuable than others (Strauss & Francis, 2017). Claims that listeners strategically allocate their cognitive resources during speech perception (Peelle, 2018) imply the presence of powerful mechanisms that direct the phonetic recalibration process—perhaps hinging on the listener’s locus of perceptual attention (Mirman et al., 2008) and/or motivation (Herrmann & Johnsrude, 2020). Attempts to modulate attention have, in limited cases, been shown to affect lexically guided perceptual learning. Learning is attenuated or even blocked entirely when listeners must simultaneously attend to multiple auditory streams, such as via tasks demanding attention to other voices, environmental sounds, or pure tones (Jesse & Kaplan, 2019; Samuel, 2016). This finding is likely attention-related rather than a result of auditory masking, as learning reemerges when the same auditory distractions are present but the task does not require attention to them (Samuel, 2016). Directly alerting listeners to the ambiguity of a talker’s productions has also numerically reduced phonetic recalibration effects (McAuliffe & Babel, 2016), suggesting that shifts in listener attention play a role in lexically guided perceptual learning.

Aside from the examples above, attempts to manipulate talker-specific learning via modulating attention have been largely unsuccessful. Lexically guided perceptual learning is robust across cognitive load and unaffected by concurrent tasks such as remembering object paths or sequences of letters (Baart & Vroomen, 2010; X. Zhang & Samuel, 2014). Learning is also unaffected by the attractiveness of a talker’s voice (Babel, Senior, et al., 2019) and is not attenuated by tasks that are difficult enough to interrupt comprehension of connected speech (X. Zhang & Samuel, 2014). The robust and rather unflappable nature of lexically guided perceptual learning is especially striking given that effects are rapid, sophisticated, and specific. Learning emerges after as few as four tokens of input (Cummings & Theodore, 2022) and reflects reshaping of sound–meaning correspondences beyond simple boundary shifts (Clarke-Davidson et al., 2008; Xie et al., 2017); additionally, listeners can adapt perception to multiple talkers with different phonetic signatures within the same session, albeit potentially with higher demands in terms of exposure extent (Cummings & Theodore, 2023; Kraljic & Samuel, 2007; Luthra et al., 2021a, 2021b). This constellation of findings suggests that lexically guided perceptual learning itself, as well as its relationship to attention, is highly complex and context-specific.

These mixed findings regarding whether or not attentional manipulations affect perceptual learning of speech stimuli motivate the current study. Specifically, we aim to manipulate value-driven selective attention while listeners learn the phonetic idiosyncrasies of two talkers. Though listeners are shown to be able to update their beliefs about the phonetic signatures of two talkers simultaneously (Cummings & Theodore, 2022; Luthra et al., 2021a, 2021b), it remains unclear whether attention can modulate the phonetic recalibration effect for each talker. If we find that an extrinsic reward drives greater perceptual learning for the higher-value talker, then we can conclude that listeners use value-derived cues, when they are available, to selectively prioritize the speech input from one talker over another. On the other hand, if listeners do not engage in value-directed attentional capture during lexically guided perceptual learning, then it remains to be explored as to why perceptual learning in the speech modality may be more insulated from domain-general influence than other sensory and perceptual modalities (Itthipuripat et al., 2019; Kim et al., 2021; Mine & Saiki, 2015; Sanz et al., 2018). As of yet, models that endeavor to capture how phonetic recalibration occurs have not formally included value-based and/or motivational cues in their computational architectures (Kleinschmidt & Jaeger, 2015; Mirman et al., 2006). However, some models of perceptual learning have posited a role for attention, raising the tantalizing possibility that broad features of the listening environment may modulate attention, potentially shaping learning (Strange, 2011; Xie et al., 2023).

In the current study, we paired multi-talker lexically guided perceptual learning (Cummings & Theodore, 2022; Luthra et al., 2021a, 2021b) with reward-based associative learning (Anderson, 2016a, 2016c) to test whether talker-specific learning is sensitive to reward-directed shifts in selective attention. Notably, we implemented a probabilistic reward schedule wherein listeners were not told explicitly that one talker was more rewarded than the other. The implicit reward manipulation resulted in one talker being rewarded (with a small monetary incentive on the order of a few cents per trial) three times as often as the other, leading to the emergence of a “high-reward” talker and a “low-reward” talker. This choice was motivated in part by findings in the lexically guided perceptual learning literature showing impoverished learning when listener attention was explicitly drawn to the manipulation, such as notifying them to attend to the ambiguous phoneme (McAuliffe & Babel, 2016). Following this logic, if listeners were told explicitly which talker was the high-reward talker, it could cause them to disregard the phonetic information altogether resulting in no learning effect for either talker. Further, work outside of speech perception has shown that awareness of the rewarded stimuli is not needed to drive attentional perceptual shifts (Seitz & Watanabe, 2009). The probabilistic nature of the reward manipulation also echoes the complex nature of typical social contexts in which listeners must track multiple sources of information at once, often without explicit cues as to what’s important (Qian et al., 2012). In a dynamic and demanding world, listeners may learn to use a variety of emergent cues to direct their limited attentional resources towards talkers that hold the most value for them.

In previous research, monetary reward has been used to drive postperceptual shifts in categorization of speech stimuli. In one such study (Connine & Clifton, 1987), listeners were asked to categorize stimuli from a nonword–nonword continuum (e.g., *dicel*–*ticel*). Critically, for ambiguous stimuli, one answer (e.g., *dice*) was associated with a high monetary payoff and the other with a high monetary penalty. (For unambiguous stimuli, the acoustically congruent answer was always rewarded more than the acoustically incongruent one.) This reward schedule induced a shift in the category boundary, but no effect on reaction times was observed at the boundary. The authors contrasted categorization shifts driven by reward and those driven by lexical knowledge, since lexical knowledge not only drives shifts in categorization (e.g., listeners are more likely to categorize ambiguous stimuli from a *dice*–*tice* continuum as *dice*) but also induces a reaction time advantage for lexically consistent interpretations at the category boundary; the differential effects of monetary reward and lexical knowledge on reaction times was taken as evidence that these factors can influence speech processing in different ways. However, evidence that reward can modulate phonetic categorization in a postperceptual way does not necessarily mean that it cannot also shape perceptual processing. Sentence context, for example, can drive postperceptual shifts in phonetic categorization (as evidenced by reaction time data; Connine, 1987) but can also drive perceptual learning for speech sounds (Jesse, 2021). In the current work, we ask whether monetary reward can modulate how lexical information shapes perceptual processing; we hypothesize that reward can bias listeners’ attention toward one talker, potentially enhancing perceptual learning.

In our experiments, participants made decisions either about the identity of the talker or the identity of the phonetic category; however, in either case, we do not expect our design to induce meaningful response biases. In the case of the talker decision task, the two talkers are highly distinct, whereas we would only expect potential response biases to emerge on stimuli where the talker identity is ambiguous. In the phonetic categorization task, the dimension being rewarded (talker) is distinct from the task-relevant dimension (phonetic category). Additionally, the critical learning trials during exposure are lexically disambiguated regardless of reward, and previous study has shown additional inclusion of postperceptual influences (such as sentential context) not to affect lexically guided perceptual learning (Luthra et al., 2021a, 2021b). We therefore do not predict reward to affect learning at the level of an individual trial. Rather, over the course of exposure as a whole, listeners may learn the association between a given talker and the reward linked to that talker.

Within a single talker, learning has been shown to be exquisitely sensitive to situational details such as language or articulatory context (Caudrelier et al., 2024; Kraljic et al., 2008). Our hypothesis, that reward associated with a talker may affect learning, is worth conditioning on previous work showing indexical factors of talkers not to interact with learning. For example, listeners exposed to an unattractive versus attractive voice do not differ in extent of demonstrated learning (Babel et al., 2019a, 2019b). However, this manipulation was between subjects, such that no listener heard both voices. It remains plausible that these sorts of features could beget differences given the potentially higher attentional demands of simultaneous exposure to multiple talkers, particularly with our stimulus set (see Luthra et al., 2021a, 2021b). We ask then, given monetary value’s previously established influence on category decisions and selective attention, whether talker “value” in an explicit monetary sense may function as such a situational detail and therefore modulate lexically guided perceptual learning.

Using a multi-talker lexically guided perceptual learning paradigm, we conducted five experiments to test whether adaptive processes in speech perception are subject to changing circumstances in the listening environment, such as perceived talker value. We hypothesized that if lexically guided perceptual learning is sensitive to reward-directed attention to a talker’s voice, we should see larger phonetic recalibration effects for the high-reward talker compared with the low-reward talker. We tested this hypothesis under multiple different iterations of the reward manipulation: probabilistic and deterministic, pre- and postdecision during the exposure task, and with two different exposure tasks. We have organized these manipulations under three overarching questions and a series of post hoc analyses, which we will address in turn.

Question 1 asks whether differences in the magnitude of the reward associated with each talker (high versus low) results in corresponding changes in the magnitude of the bias effect. We conducted two experiments for Question 1. Experiment 1A implements a probabilistic reward schedule during the exposure cover task while Experiment 1B substitutes that reward for a simple visual stimulus to test whether any effect on phonetic categorization can be attributed to reward-mediated attentional biases or simple low-level attentional biases. Further, Experiments 1A and 1B use a talker-decision exposure task that focuses listener attention at the talker level.

Under Question 2, we examine whether linking the reward to a different property of the stimulus during exposure (the phoneme) improves transfer to the phonetic categorization task. Previous work has established that perceptual learning does not always generalize across stimulus dimensions (Fahle, 2005; Schlegelmilch & von Helversen, 2020; Seitz et al., 2023; but see Drouin & Theodore, 2018), raising the possibility that a reward targeting one dimension (e.g., talker) may not modulate perceptual learning of a different dimension (e.g., phonetic detail). As such, Experiment 2A introduces an exposure task in which listeners made responses based on phonemic detail. Experiment 2A establishes whether listeners show talker-specific phonetic recalibration after a phoneme-monitoring exposure task, while Experiment 2B implements the probabilistic reward schedule with the updated task to test if the size of the bias effect changes as a result of the value of the talker.

Question 3 tests whether explicit awareness of the value of each talker is necessary to induce differences in the magnitude of the bias effect. It is possible, considering the limited number of trials by which to learn the association between each talker and the reward magnitude, that listeners may not have the time to engage with value-driven shifts in selective attention. Thus, in Experiment 3, we gave participants a reward cue prior to the onset of the auditory token during the exposure cover task that indicates whether the upcoming talker is of high value or low value. We also provided the reward cue on every trial (i.e., a deterministic cue) to give listeners the best possible chance of selectively attending more so to the high-value talker. Ultimately, the purpose of the current study is to gain insight into how socially relevant cues like perceived value may bias the selective attention of listeners to modulate the degree of lexically guided perceptual learning for multiple talkers.

## General methods

### Stimuli

The auditory stimuli in the current study were developed and used in previous studies (Luthra et al., 2021a, 2021b). Identical stimuli for the exposure and phonetic categorization tasks were used in all experiments reported here. In brief, there were 32 unique words heard by participants during the exposure task. Sixteen words had a medial /s/ (e.g., *episode*) and the other 16 had a medial /ʃ/ (e.g., *impatient*). A female speaker of North American English produced the original tokens, which were then normalized to 70 dB SPL. The ambiguous fricatives were created by blending clear endpoints of each word (e.g., *dinosaur* and *dinoshaur*) using the STRAIGHT software (Kawahara, 2006) then re-inserting the blended fricative back into the word frame (i.e., *dino?aur*, where the ? represents the ambiguous s/sh blended token). The finalized female stimuli were gender-shifted using the “Change Gender” tool in Praat (Boersma & Weenink, 2017) to create the “male” tokens. Informal piloting suggested that this manipulation was successful, since lab members who knew the model talker personally were convinced that the gender-shifted voice was a new talker. Stimuli were organized into “/s/-biasing” and “/ʃ/-biasing” lists and, during exposure, listeners heard both male and female talkers, with each talker corresponding to a different biasing direction. The “s-biasing” list contained clear versions of the /ʃ/ words (e.g., *impatient*) and blended versions of the /s/ words (e.g., *dino?aur*), whereas the “/ʃ/-biasing” list contained clear versions of /s/ words (e.g., *dinosaur*) and blended versions of the /ʃ/ words (e.g., *impa?ent*).

The phonetic categorization task included tokens in a 7-step continuum from a clear *sign* to a clear *shine* that were created using the same procedure as the exposure task stimuli. For more details about the auditory stimuli, see Luthra et al., (2021a, 2021b).

## Experimental design

**Fig. 1:**
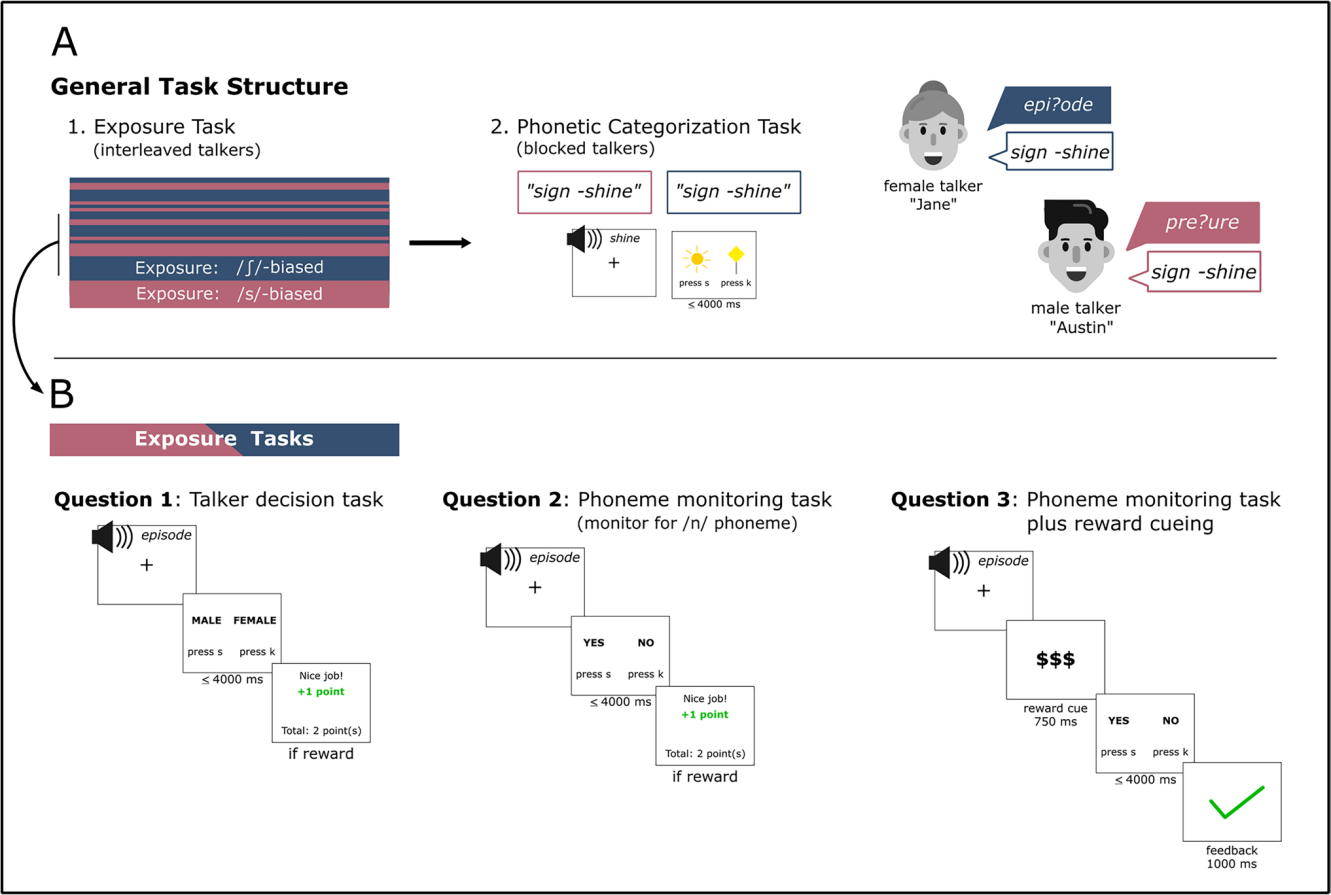
**A** Schematic of the experiment task structure. All experiments had the same task progression. In the exposure phase, participants heard tokens from both talkers, presented in an interleaved fashion. The exposure task varied slightly for each experimental question (see Panel B). After the exposure task, participants immediately completed a phonetic categorization task which was blocked by talker; talker order was counterbalanced across participants. The phonetic categorization task was a 2AFC decision where participants indicated by button press whether a given token on a 7-point continuum from *sign-shine* was more like “sign” or like “shine.” **B** Variants on the exposure task by experimental question. Experiments 1A and 1B used a talker decision task where participants indicated whether the talker was male or female and received feedback and a reward (points that corresponded to a small monetary payout) for correct answers on a subset of the trials. Experiments 2A, 2B, and 3 transitioned to a phoneme monitoring task where participants indicated if the auditory word contained the “n” phoneme or not. Again, they received feedback and a reward for a subset of correct responses. Experiment 3 used the same phoneme monitoring task but added a reward cue prior to the auditory stimulus that indicated the value of the talker. After the phoneme monitoring response, participants also received feedback. (Color figure online)

Each experiment in the current study had the same two-part structure (see Fig. 1A). Manipulations specific to each research question occurred only in the exposure task and will be described in depth within each question subsection (see Fig. 1B for overall schematic). During the exposure task, participants listened to the male and female talkers produce 32 unique words—16 unambiguous (i.e., clear word-medial fricative) and 16 ambiguous (i.e., blended word-medial fricative)—while looking at a central fixation. For the ambiguous tokens, each talker was biased in a different direction, such that one talker’s lexical context biased the interpretation of the ambiguous medial fricative toward an /ʃ/ interpretation and the other talker biased the interpretation of the ambiguous token toward /s/ (see Stimuli, above, for examples). We counterbalanced the bias direction of each talker across participants such that, for instance, a participant would only hear the /s/-biased female talker and the /ʃ/-biased male talker during exposure while another participant would hear the reverse (/ʃ/-biased female and /s/-biased male talker). We used two different tasks during exposure—talker decision and phoneme monitoring—that will be described in more detail in subsequent sections. For both exposure tasks, participants were given 4,000 ms to make a two-alternative forced-choice (2AFC) decision using the “k” and “s” buttons on the keyboard. Feedback was provided immediately following responses and was displayed for 1,000 ms. The feedback timing was adopted from work in speech category learning that has shown that immediate feedback improved learning while delayed feedback (feedback given after an interval of 500–1,000 ms) slowed learning (e.g., Chandrasekaran et al., 2014). Following feedback, there was an interstimulus interval of 1,000 ms before the onset of the next auditory token. We counterbalanced the order of the key-mapping of the poststimulus decision across participants. Based on findings from previous work using this task design (Luthra et al., 2021a, 2021b; but see Cummings & Theodore, 2022), we presented each unique exposure trial twice, such that participants heard 128 total trials; 64 trials for each talker (32 unique words presented twice each).

Immediately following the exposure task, participants completed a phonetic categorization task that was identical across all experiments reported here. For each talker, participants heard 10 repetitions each of a seven-step continuum from *sign* to *shine*. We blocked this task by talker, such that there were 70 total tokens for the female talker and 70 for the male talker. The order of the blocks was counterbalanced across participants, and trial order was randomized within each block. In the task, participants indicated via 2AFC whether they believed the token they heard was the word *sign* or *shine*. They had 4,000 ms to respond. The position of the *sign* and *shine* images on the screen and their corresponding key mappings were counterbalanced across participants.

## Experiment procedure

All experiments were programmed and delivered on Gorilla Software Builder (Anwyl-Irvine et al., 2020), and participants were recruited online via Prolific (Palan & Schitter, 2018). We screened participants using the following parameters to maximize data quality: current IP address located inside the United States, between 18 and 90 years of age, normal vision, monolingual English speakers, no language disorders, no hearing difficulties, using a desktop computer, and a Prolific approval rating of 90–100. Participation was limited to one experiment, and they could not have participated in any other experiments that have used the same stimuli and task design (Heffner et al., 2022; Luthra et al., 2021a, 2021b). Per the Institutional Review Board at the University of Connecticut, all participants gave consent via a digital information sheet and were given a base payment of $10/hour (average of $3.33 since the study took approximately 20 min). For the three experiments that included a reward component, participants were also given a bonus payment equivalent to their performance on the exposure task. This bonus compensation did not exceed $2.56 but did vary slightly for each experiment.

Once participants gave consent, they performed a headphone screener (Milne et al., 2021) to test whether participants were wearing headphones. The design of the screener leads to poor performance if participants are listening over speakers versus headphones or earbuds. Participants were given two chances to pass the headphone screener and were reminded to put on headphones after the first failure. While participants who failed the screener twice were allowed to complete the full task, they were excluded for all data analyses. Afterward, participants answered a short demographics questionnaire that asked for sex, age, ethnicity, race, and regional accent. They then completed the exposure task and then the phonetic categorization task. At the end of the experiment, participants were told what the purpose of the study was and were told the amount of their bonus compensation. Prior to data analysis, we excluded participants based on the following criteria: two failed headphone screeners, failure to respond to 10% of the trials in either the exposure or phonetic categorization tasks, and/or less than 70% coherence on endpoint decisions during phonetic categorization. Further, a small number of participants were randomly excluded to maintain an approximately equal number of participants in each counterbalancing condition.

## Data analysis

We analyzed the data from the phonetic categorization task using linear mixed-effects models in R (Version 4.1.1; R Core Team, 2021) using the *glmer* function from the “lme4” package (Bates et al., 2015). Fixed factors included continuum Step (seven steps between *shine* to *sign;* centered using the *scale* function), Bias (deviation coded [− 0.5, 0.5], /s/-bias, /ʃ/-bias), and Rewarded Talker [deviation coded [− 0.5, 0.5], low-reward talker, high-reward talker). To note, one experiment, Experiment 2A, did not include a probabilistic or reward manipulation and thus the Rewarded Talker fixed factor was not included in the model. We used a backward-stepping procedure to determine the random-effects structure (Matuschek et al., 2017), and in all cases the maximal model (Barr et al., 2013) was the best-fitting model as evaluated using a likelihood ratio test. Of note, while both Rewarded Talker and Bias were manipulated within subjects, they were always perfectly correlated (i.e.., a given listener never heard the same talker with both high and low reward). For this reason, the maximal random effects structure licensed by our design does not include the interaction of random slopes by subject for rewarded talker and for bias. All data and code are openly available online (https://osf.io/y52ge/).

## Question 1: Does monetary reward affect talker-specific phonetic recalibration?

To probe if listeners show a larger bias effect for a talker that receives more reward than another talker, we implemented a probabilistic reward schedule during the exposure task and then subsequently tested the magnitude of the bias effect for each talker via a 2AFC phonetic categorization task. For the two experiments reported here, the exposure cover task required participants to make decisions about the gender identity of each talker. In the reward manipulation included in Experiment 1A, a subset of the trials for each talker involved a reward; participants were either awarded a single point for a correct response or given no points for an incorrect response. For the high-reward talker, the probability of receiving a reward was set at 37.5% of trials in the exposure task while the low-reward talker only had the probability of receiving a reward on 12.5% of trials. At the end of the study, the number of points participants accumulated were converted to a proportional monetary bonus. We expected that if participants were sensitive to the reward manipulation and accordingly shifted their attention to the high-reward talker at the expense of the low-reward talker, then we would see a larger bias effect at test for the high-reward talker.^1^

We also ran a second experiment, Experiment 1B, that controlled for any low-level attentional effects. As participants in Experiment 1A were three times as likely to see a screen containing feedback and rewards for one talker compared with the other, we wanted to ensure that any bias effects that emerged were due to the reward and not due to seeing low-level visual information more often for one talker. To control for this potential confound, we exchanged the reward/feedback screen for a neutral shape using the same probabilistic schedule. For the “high-reward” talker, participants had the probability of seeing a square following correct responses or a circle following incorrect responses on 37.5% of trials. For the “low-reward” talker, participants saw a square or circle on only 12.5% of trials. We predicted that we would find no variation in the magnitude of the bias effect for the “high-reward” and the “low-reward” talkers, as neutral, low-level visual information is likely insufficient to bias listeners towards selectively attending to one talker versus the other.

### METHOD

## Procedure

Exposure trials for Question 1 are schematized in Fig. 1B. The exposure task consisted of 128 randomly intermixed trials; 64 for the male talker and 64 for the female talker. The cover task for Experiments 1A and 1B was a talker decision task in which participants, after each auditory token, indicated the gender identity of the talker via 2AFC (MALE–FEMALE). In Experiment 1A, one talker, either male or female, was designated the high-reward talker and the other the low-reward talker. The gender identity of the high- and low-reward talker was counterbalanced across participants. Thus, a participant would either have a high-reward male and low-reward female talker or a high-value female and low-reward male talker. For the high-reward talker, participants had the possibility of receiving a reward on 37.5% of trials (24 trials out of 64) while the low-reward talker only had the possibility of reward on 12.5% of trials (eight trials out of 64). On the same proportion of trials, participants received feedback as to the correctness of their response on the talker decision task. For all other trials, participants did not receive feedback or a reward. All participants then completed the phonetic categorization task as described in the general methods. At the end of the entire experiment, participants were asked if they believed the male or the female talker was rewarded more often. Answers to this prompt were included in a supplementary analysis probing whether awareness of which talker was rewarded more often predicted the bias effect.

Experiment 1B used the same cover task and probabilistic schedule as in Experiment 1A but replaced the reward and feedback for neutral shapes. Using the probabilistic schedule, correct responses were followed by a solid black square presented in the center of the screen while incorrect responses were followed by a solid black circle. These neutral visual stimuli were presented for 1,000 ms. The probability of seeing a shape following a response on the talker decision task was set at 37.5% of trials for the “high-reward” talker and 12.5% of trials for the “low-reward” talker. All participants then completed the phonetic categorization task. After the experiment concluded, we asked two follow-up questions to probe if participants 1) learned the meaning of the shapes (i.e., as abstract indications of feedback) and 2) tracked whether the square shape (corresponding to correct answers) occurred more often following tokens spoken by the male or female talker. We did not include responses to either question in any further analyses included in the current study.

## Participants

We recruited 238 participants from Prolific for Experiment 1A and 75 for Experiment 1B. To note, we collected participants in three waves for Experiment 1A.^2^ After exclusions, we had a total of 192 participants (107 women, 80 men, five unknown; mean age = 29.1 years, age range: 50 years) for Experiment 1A, and 64 participants (32 women, 32 men; mean age = 30.7 years, age range: 52 years) for Experiment 1B.

## RESULTS

**Fig. 2:**
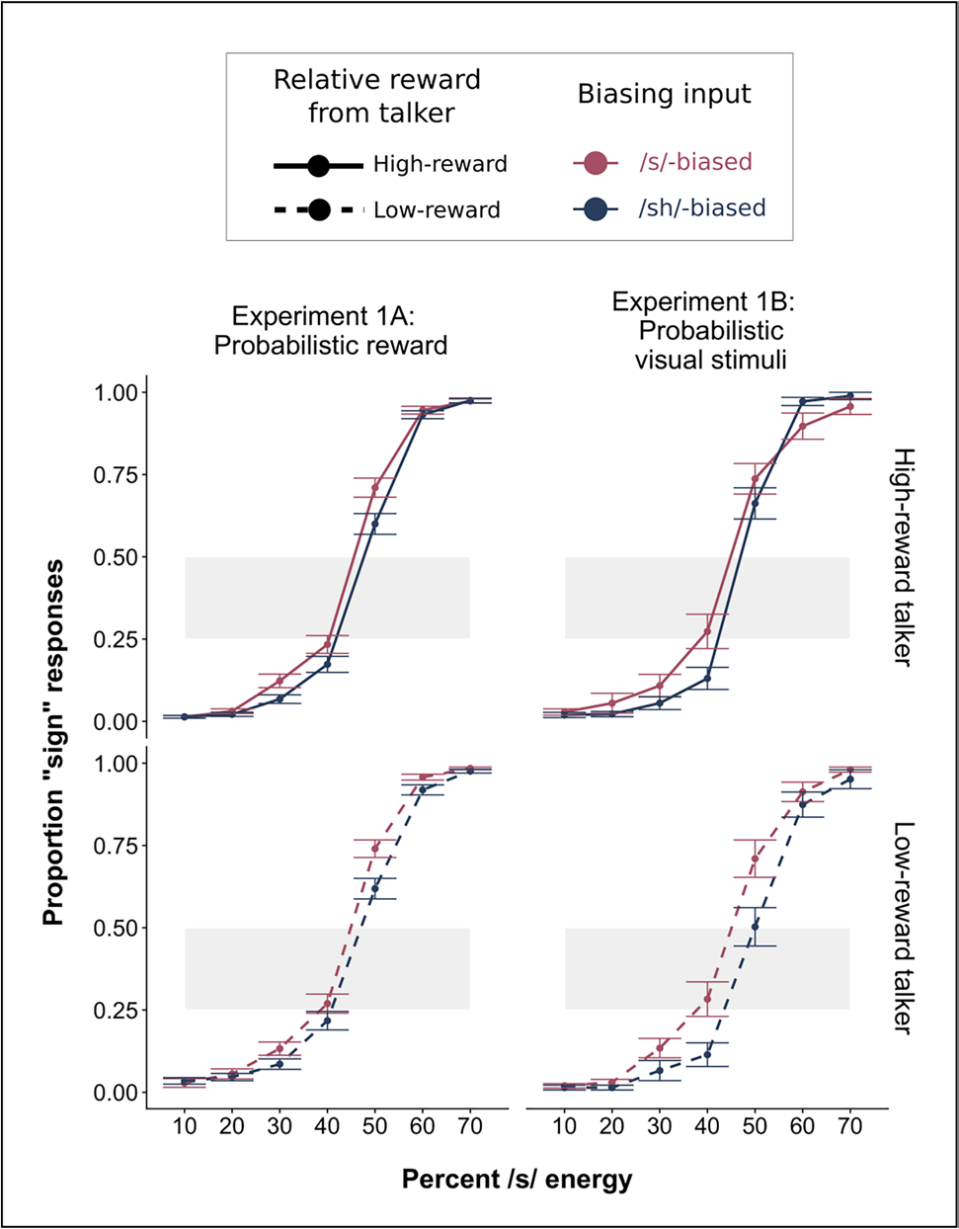
Results from the phonetic categorization task from Experiment 1A (left column) and Experiment 1B (right column). The red lines indicate categorization for the /s/-biased talker and the blue lines indicate categorization for the /ʃ/-biased talker. On the *x-*axis, we plot the acoustic energy as the proportion of /s/ energy, proceeding from more /ʃ/-like responses on the left to more/s/-like responses on the right-hand side. Shaded gray regions indicate the range depicted in Fig. 5. Rewarded Talker is indicated by line type: solid for high-reward talker and dashed for low-reward talker. (Color figure online)

Model output and model syntax for the linear regressions can be seen in Table 1A and 1B, and the plotted results for Experiments 1A and 1B can be seen in Fig. 2A. Our primary question was whether the value of the talkers during the exposure phase modulated the extent of the bias effect in the phonetic categorization task. Experiment 1A tested this hypothesis (Table 1A). The expected main effect of Step was significant (β = 4.87, *p* < 0.001), which shows that listeners reliably categorize items differently across the continuum. A main effect of Bias (β = 0.54, *p* < 0.001), reflected the predicted influence of the exposure condition, with more /ʃ/ responses to the sh-biased talker and more /s/ responses to the s-biased talker. However, the interaction of Bias and Rewarded Talker was not significant (β = 0.09, *p* = 0.836). The lack of interaction indicates that the reward manipulation did not result in corresponding changes in the magnitude of the bias effect. In other words, participants showed equivalent magnitude bias effects for the high-reward and low-reward talkers.

**Table 1:** Phonetic categorization results from Question 1

**A.** Experiment 1A: Talker decision exposure task and probabilistic reward schedule					
SH_resp ~ Step × Bias × Rewarded Talker + (Step × ( Bias + Rewarded Talker | Subject)					
**Fixed effects**	**Estimate**	**Std. error**	***Z* value**	***p* value**	**Significance**
Step	4.87	0.129	37.70	< 0.001	***
Bias	0.54	0.15	3.50	< 0.001	***
Rewarded Talker	0.26	0.15	1.86	0.064	
Step × Bias	0.042	0.17	0.25	0.80	
Step × Rewarded Talker	0.14	0.17	0.84	0.40	
Bias × Rewarded Talker	0.09	0.44	0.21	0.84	
Step × Bias × Rewarded Talker	0.40	0.52	0.77	0.44	
**B.** Comparison between Experiment 1A (talker decision with probabilistic reward) and Experiment 1B (talker decision with visual shape stimuli)					
SH_resp ~ Step × Bias × Experiment + (Step × Bias | Subject)					
**Fixed effects**	**Estimate**	**Std. error**	***Z* value**	***p* value**	**Significance**
Step	4.92	0.11	14.71	< 0.001	***
Bias	0.87	0.16	5.57	< 0.001	***
Experiment	− 0.18	0.22	− 0.81	0.419	
Step × Bias	0.50	0.17	2.92	< 0.01	**
Step × Experiment	− 0.07	0.26	− 0.26	0.79	
Bias × Experiment	− 0.56	0.31	− 1.80	0.072	
Step × Bias × Experiment	− 8.0	0.34	− 2.35	0.02	*

^a^A priori, it is possible that the third order interaction of Step, Bias, and Rewarded Talker, which accounts for significant variance in this model, is limiting the second order interaction of interest (Bias and Rewarded Talker) from reaching significance. While we abstain here from adjusting model structure to account for this possibility, exploratory analyses which further investigate the findings of Experiment 3 can be found in the Supplemental Materials associated with this manuscript

In Experiment 1B, we found expected main effects of Step (β = 4.99, *p* < 0.001) and Bias (β = 1.31, *p* < 0.001), and a significant interaction of Step and Bias (β = 1.08 *p* < 0.001). We also did not find a significant effect of the probabilistic shape manipulation either as a main effect (β =  − 0.36, *p* = 0.18) or as an interaction with Bias (β =  − 1.11, *p* = 0.14). The full results can be found in the Supplementary Materials (Supplementary Table 2).

To test whether differences in the phonetic categorization task could be attributed to low-level attentional shifts induced by the mere fact that participants saw more visual content (the feedback screen) associated with one talker than the other, we included Experiment 1A (the reward manipulation) and Experiment 1B (control manipulation using visual shapes rather than feedback) in the same model (Table 1B). We found that the interaction between Bias and Experiment approached significance (β =  − 0.56, *p* = 0.0719); however, this marginal effect would indicate less learning in the reward manipulation than the neutral manipulation. We refrain here from overinterpreting a small and marginal effect and return to this question in post hoc summary analyses (see Fig. 6).

Model syntax on Line 2. Significance is indicated by asterisk number adhering to classic convention: *p* < 0.001 (***), *p* < 0.01 (**), *p* < 0.05 (*), *p* > 0.05 ( +), marginal significance (.)

## Question 2: Does the exposure task influence the sensitivity of phonetic recalibration to talker value?

The results of Experiments 1A and 1B were unexpected, in that 1) participants did not show variation in the size of the bias effect contingent on talker value and 2) learning may have actually been attenuated in the reward manipulation when compared with the neutral control version of the probabilistic task. Yet it is possible that the nature of the exposure task interfered with potential reward effects. Specifically, in Experiment 1A participants were rewarded for correct decisions on the talker gender decision. Focusing attention at this indexical level of processing might limit the influence of reward on phonetic recalibration. For Question 2, we altered the exposure task to draw participant attention to the phonetic details of the auditory tokens. To that end, we designed a phoneme monitoring task in which participants indicated whether or not the auditory token contained the /n/ phoneme or not. Lexically guided perceptual learning tasks have successfully shown phonetic recalibration using a number of different tasks during the exposure phase including lexical decision (Clarke-Davidson et al., 2008), story listening (Eisner & McQueen, 2006; Maye et al., 2008), syllable counting (Samuel, 2016), amplitude monitoring (Drouin & Theodore, 2018) and talker decision (Luthra et al., 2021a, 2021b) though none have used phoneme monitoring to the best of our knowledge. Experiment 2A first validates that participants are able to learn the phonetic details of two talkers simultaneously using the phoneme monitoring cover task, while Experiment 2B re-implements the probabilistic reward schedule described in Question 1 where we test, again, if the bias effect is modulated by the value of the talkers during exposure.

### METHODS

## Procedure

See Fig. 1B under “Question 2” for a schematic of the phoneme monitoring cover task. In this task, participants needed to indicate via a YES–NO 2AFC if the word they just heard contained the /n/ phoneme. This phoneme occurred in 22 out of 32 tokens. We intentionally did not ask participants to monitor for a fricative phoneme, as directing attention to atypical phonetic productions has been shown to potentially diminish perceptual learning (McAuliffe & Babel, 2016). Experiment 2A assessed whether the phoneme monitoring task, without feedback or reward, results in a bias effect for each talker. Experiment 2B combined the phoneme monitoring task with the probabilistic reward schedule to test if value-induced shifts in attention to a rewarded talker results in changes in the magnitude of the bias effect at test. We used the same probabilistic schedule as described in the Procedure for Question 1, where the high-reward talker had the possibility of a reward on 37.5% of trials and the low-value talker had the possibility of reward on 12.5% of trials.

After completing the phoneme monitoring cover task during exposure, all participants then completed the phonetic categorization task for each talker. Talker order was counterbalanced across participants. Upon the conclusion of both tasks in Experiment 2B, participants were informed of the magnitude of the monetary bonus they earned for their performance on the phoneme monitoring task, which did not exceed $2.56. Finally, participants in Experiment 2B were asked whether they knew which talker, male or female, was rewarded most often. We did not use responses to this question in analyses reported here.

## Participants

For Experiment 2A, we recruited 34 participants from Prolific and from the University of Connecticut’s Psychology Department participant pool. After exclusions, 16 participants remained from the online pool of participants and 16 from the University of Connecticut pool resulting in a total of 32 participants (19 women, 12 men, one unknown; age range: 18–42 years,^3^ mean = 23). For Experiment 2B we exclusively recruited 112 participants from Prolific. After exclusions, 64 participants (31 women, 31 men, two unknown; age range: 20–46 years, mean = 32) remained for data analyses.

## RESULTS

**Fig. 3:**
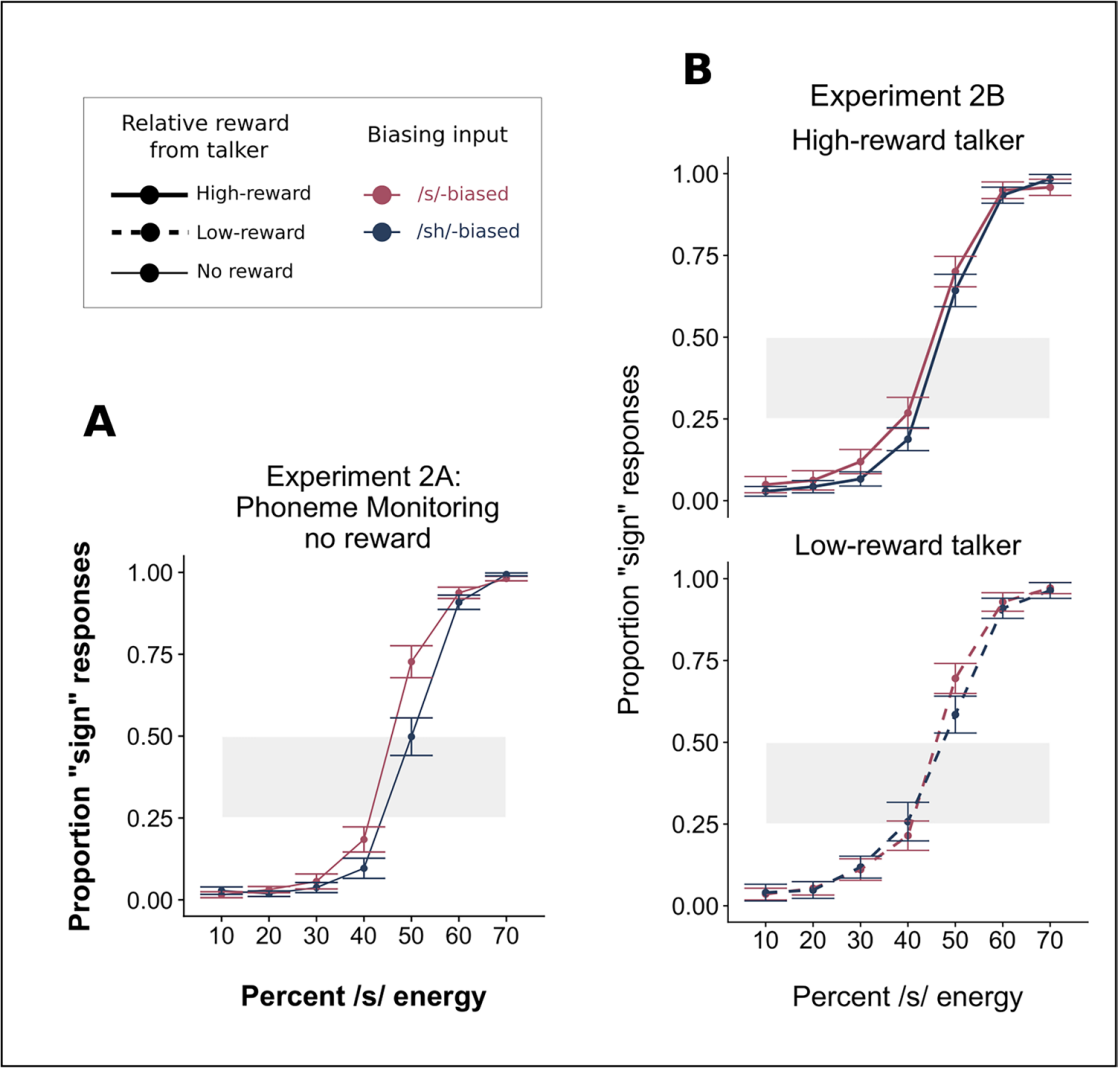
Results from the phonetic categorization tasks in Experiment 2A (**A**) and Experiment 2B (**B**). The biasing direction is indicated by color; red corresponds to the /s/-biased talker and blue indicates the /ʃ/-biased talker. On the *x*-axis, we plot the acoustic energy as the proportion of /s/ energy, proceeding from more /ʃ/-like responses on the left to more /s/-like responses on the right-hand side. Shaded gray regions indicate the range depicted in Fig. 5. **B** Results for Experiment 2B, where the magnitude of reward associated with each talker during exposure is indicated by line type, with solid lines indicating responses for the high-reward talker and dashed lines indicating the low-reward talker. (Color figure online)

Plotted results can be seen in Fig. 3 for the phonetic categorization tasks in Experiment 2A (3A) and 2B (3B), and model outputs and model syntaxes for the linear regressions are listed in Table 2. For Experiment 2A, we tested whether participants showed talker-specific phonetic recalibration when we used a phoneme-monitoring cover-task (Table 2A). There were expected main effects of Step (β = 5.44, *p* < 0.001) and of Bias (β = 1.36, *p* < 0.001), indicating that participants showed phonetic recalibration specific to each talker and validating the continued use of the phoneme-monitoring task.

**Table 2:** Phonetic categorization results from Question 2

**A.** Experiment 2A: Phoneme monitoring exposure task with no reward					
SH_resp ~ Step × Bias + (Step × Bias | Subject)					
**Fixed effects**	**Estimate**	**Std. error**	***Z* value**	***p* value**	**Significance**
Step	5.44	0.25	8.37	< 0.001	***
Bias	1.36	0.37	3.70	< 0.001	***
Step × Bias	0.71	0.45	1.58	0.114	
**B.** Experiment 2B: Phoneme monitoring task with probabilistic reward					
SH_resp ~ Step × Bias × Rewarded Talker + (Step × (Bias + Rewarded Talker | Subject)					
**Fixed effects**	**Estimate**	**Std. error**	***Z* value**	***p* value**	**Significance**
Step	5.34	0.29	18.29	< 0.001	***
Bias	0.35	0.25	1.43	0.152	
Rewarded Talker	− 0.22	0.22	− 0.91	0.362	
Step × Bias	0.01	0.23	0.03	0.98	
Step × Rewarded Talker	− 0.21	0.23	−0.91	0.362	
Bias × Rewarded Talker	0.33	0.91	0.34	0.719	
Step × Bias × Rewarded Talker	0.41	1.17	0.35	0.73	

Model syntax on Line 2. Significance is indicated by asterisk number adhering to classic convention: *p* < 0.001 (***), *p* < 0.01 (**), *p* < 0.05 (*), *p* > 0.05 ( +), marginal significance (.)

When we combined the phoneme-monitoring task with the probabilistic reward schedule in Experiment 2B (see Fig. 3B; Table 2B), we found a main effect of Step (β = 5.34, *p* < 0.001) but no significant main effects of Bias (β = 0.35, *p* = 0.152) or of Rewarded Talker (β =  − 0.22, *p* = 0.362). We also did not find a significant interaction between Bias and Rewarded Talker (β = 0.33, *p* = 0.719). A lack of a main effect of Bias suggests that, within this sample, there is not strong evidence of phonetic recalibration for either talker.^4^ The lack of an interaction further shows that Rewarded Talker did not modulate the size of the learning for either talker.

## Question 3: Does the timing of reward influence talker-specific phonetic recalibration?

The results of Experiments 1A and 2B suggest that listeners are unpersuaded by the differential rewards associated with each talker, as the magnitude of the phonetic recalibration effect during test has either been equivalent across the high-reward and low-reward talkers (Experiment 1A) or has disappeared entirely (Experiment 2B). These results run in opposition to findings that point to a powerful role for reward in directing selective attention during perceptual tasks (for reviews, see Anderson, 2016b; Failing & Theeuwes, 2018). A possible explanation for our pattern of results is that perhaps listeners, in the course of only 128 trials during exposure, were unable to learn fast enough which talker was rewarded more often to successfully direct their perceptual attention to the higher value talker. Another possibility is that the timing of the reward could have disrupted value-directed selective attention during the exposure task. In Experiments 1A and 2B, the reward was given after participants heard the auditory token and made the cover task decision. Perhaps listeners need to be aware *prior* to the onset of the auditory token if it will be spoken by a high-reward or low-reward talker to prospectively engage attention. In Question 3, we reconfigured the phoneme-monitoring task to make the reward signal as salient as possible to test if reward is able to alter the magnitude of the phonetic recalibration task. To do this, we shifted the reward to occur prior to the onset of the word, so that listeners are cued to the value of the talker before they hear the word. Further, we eliminated the probabilistic aspect of the reward such that listeners received a reward indication and feedback on every trial. We maintained the high- and low-reward aspect, so one talker received 3 × the monetary reward value of the other talker, resulting in both talkers being rewarded, but to different degrees. We predicted that participants would show a larger bias effect for the high-reward talker than the low-reward talker.

### METHODS

## Procedure

Exposure trials for Question 3 are schematized in Fig. 1B. Participants completed the phoneme-monitoring task described under “Question 2” procedure, but with several modifications to the reward schedule. Participants were alerted in the instructions that some trials would be worth more than others. Prior to the onset of the auditory token, participants saw a reward cue for 750 ms. For the high-reward talker, participants saw “$$$” presented in the center of the screen and for the low-reward talker they saw “$.” After the offset of the auditory token, participants indicated if that word contained the /n/ phoneme via a YES–NO 2AFC. Participants were then given feedback as to the correctness of their decision with a large green check mark indicating correct responses and a large red “x” indicating incorrect responses. The reward cue and feedback were given on all trials during the exposure phase of the experiment. The gender identity of the high- and low-reward talkers were counterbalanced across participants.

Participants then completed the phonetic categorization task for both talkers, the order of which was counterbalanced across participants. After the experiment concluded, participants were debriefed and given their monetary bonus scaled to their performance on the exposure task. The maximum bonus participants could receive if they responded correctly on all 128 trials was $1.97.

## Participants

For Experiment 3, we collected 104 participants through Prolific. After exclusions, we were left with 64 participants (52 women, 12 men; age range: 18–58 years, mean = 27 years) for all analyses.

## Results

**Fig. 4:**
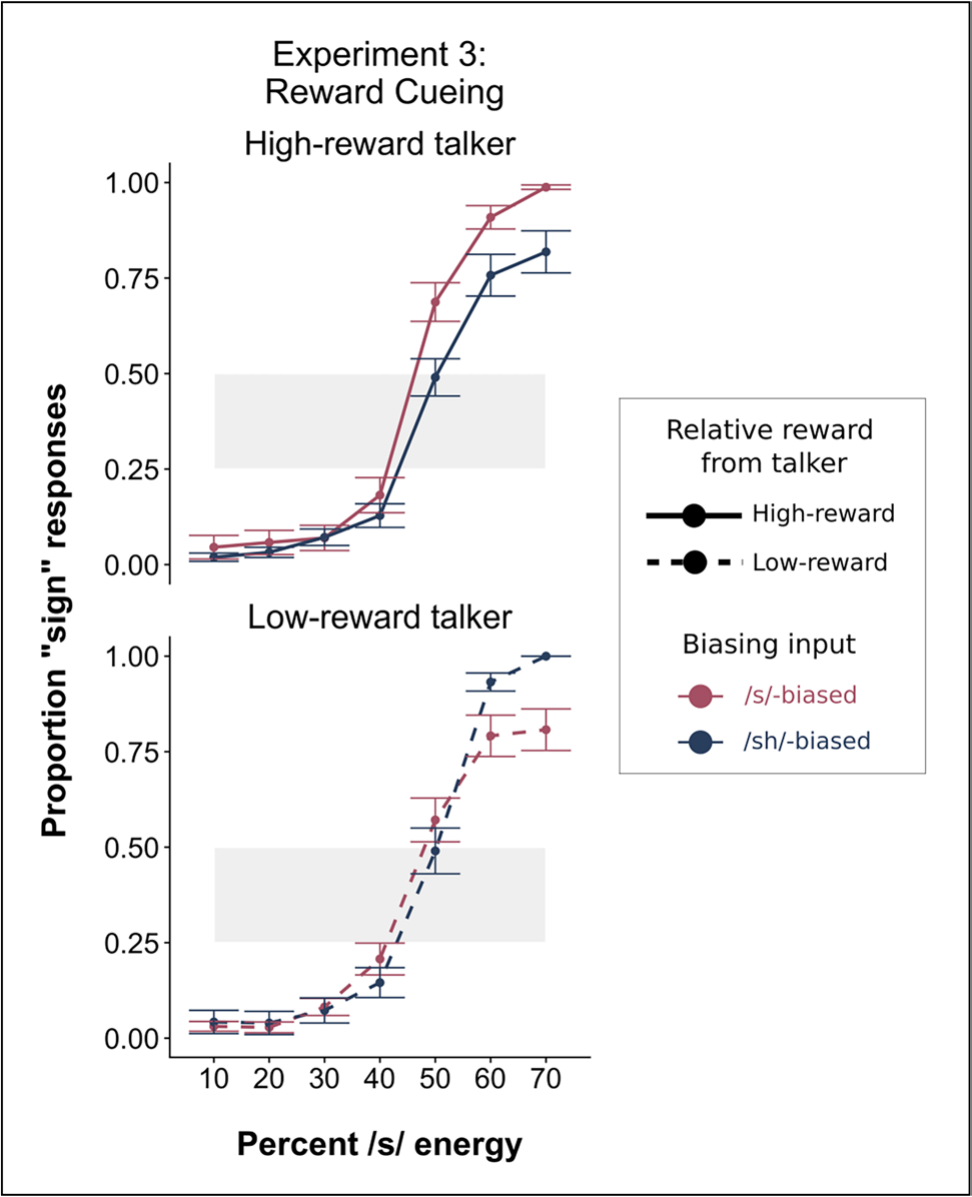
Results from the phonetic categorization task in Experiment 3. The red lines indicate categorization for the /s/-biased talker and the blue lines indicate categorization for the /ʃ/-biased talker. On the *x*-axis, we plot the acoustic energy as the proportion of /s/ energy, proceeding from more /ʃ/-like responses on the left to more /s/-like responses on the right-hand side. Shaded gray regions indicate the range depicted in Fig. 5. The magnitude of reward associated with each talker during exposure is indicated by line-type, with solid lines indicating responses for the high-reward talker and dashed lines indicating the low-reward talker. (Color figure online)

Results of Experiment 3 are plotted in the Fig. 4 and the model output is listed in Table 3. There were predicted main effects of Step (β = 5.01, *p* < 0.001) and of Bias (β = 0.73, *p* < 0.01), indicating that participants did show a talker-specific recalibration effect. Visually, the effect of biasing input appears to reverse between the high- and low-reward talkers. However, this is almost entirely driven by the /s/ end of the continuum. In our maximal model structure, which allowed for subjects to differ in intercepts and slopes across the phonetic continuum, there was no significant interaction of Bias and Rewarded Talker (β = 0.68, *p* = 0.538), suggesting that the magnitude of the Bias effect was not significantly modulated by the value of each talker. These results pattern with the previous two experiments (Experiment 1A and 2B) that also incorporated reward, wherein reward during exposure does not seem to impact the degree of phonetic recalibration measured at test.

**Table 3:** Phonetic categorization results from Experiment 3

SH_resp ~ Step × Bias × Rewarded Talker + (Step × (Bias + Rewarded Talker | Subject)					
**Fixed Effects**	**Estimate**	**Std. error**	***Z* value**	***p* value**	**Significance**
Step	5.01	0.29	17.42	< 0.001	***
Bias	0.73	0.27	2.75	< 0.01	**
Rewarded Talker	− 0.19	0.27	− 0.73	0.47	
Step × Bias	0.13	0.30	0.42	0.68	
Step × Rewarded Talker	− 0.024	0.30	− 0.08	0.94	
Bias × Rewarded Talker	0.68	1.10	0.62	0.54	
Step × Bias × Rewarded Talker	− 2.59	1.15	− 2.24	0.025	*^a^

Model syntax on Line 2. Significance is indicated by asterisk number adhering to classic convention: *p* < 0.001 (***), *p* < 0.01 (**), *p* < 0.05 (*), *p* > 0.05 ( +), marginal significance (.)

**Fig. 5:**
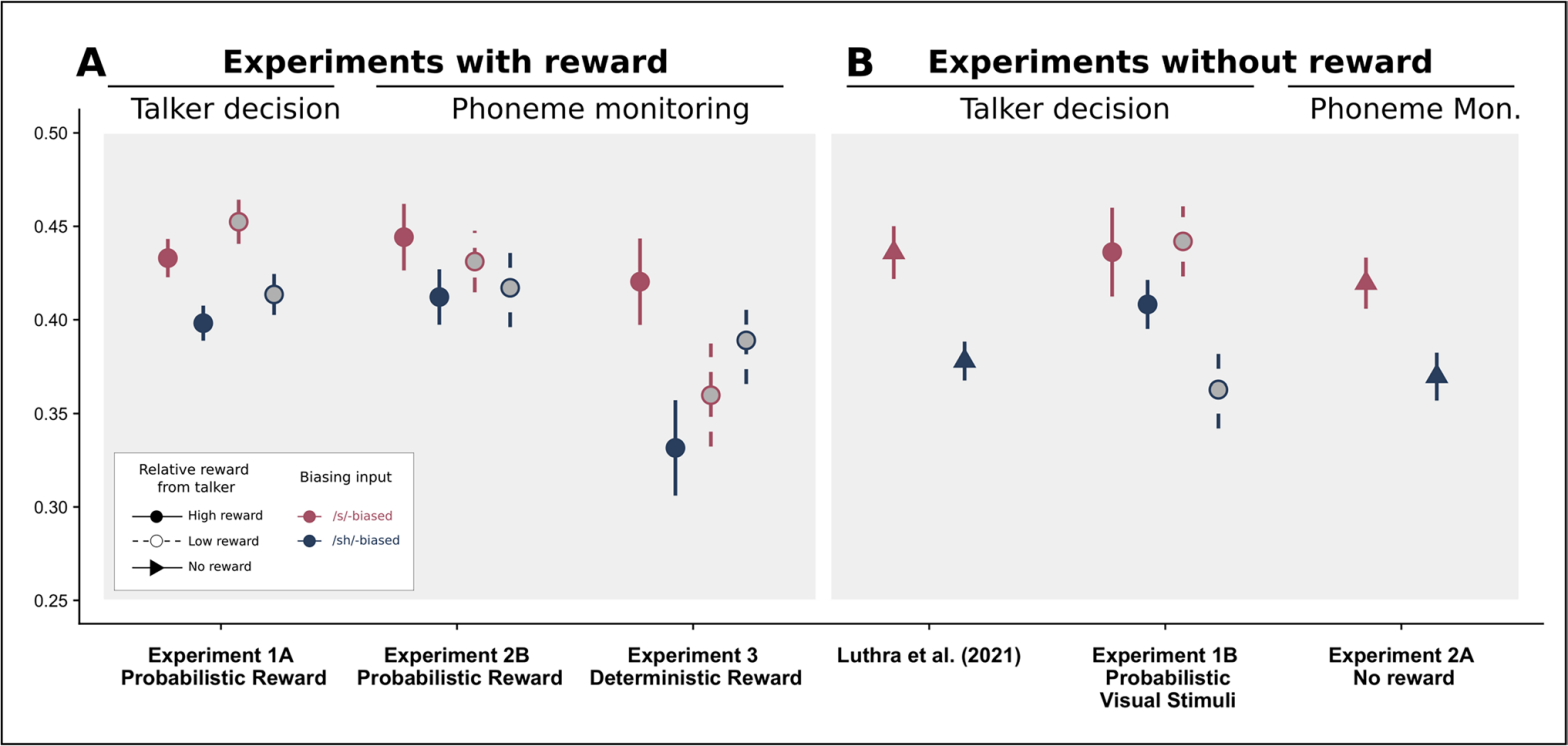
Summary figure depicting the Bias effect across all experiments. We have also included results from a previous experiment from our group using the same stimuli and paradigm (Luthra et al., 2021a) as a point of comparison. The grand mean of the /s/ responses during phonetic categorization are depicted on the *y*-axis. Bias direction is indicated by color, wherein darker colors indicate /s/-biased and lighter colors indicate /ʃ/-biased. Line length represents standard error of the mean. **A** Experiments that include reward: Experiments 1A, 2B, and 3. Rewarded Talker is indicated by line type, where solid lines are the high-reward talker and dashed lines indicate the low-reward talker. **B** Experiments that do not include reward: Data reproduced from Luthra et al., (2021a), Experiment 2B, and Experiment 2A. A reminder that in Experiment 1B, participants saw a neutral visual cue (a shape) following one talker more than the other. We have opted to display these using the same legend as the high- versus low-reward conditions

## Post hoc summary analyses

**Fig. 6:**
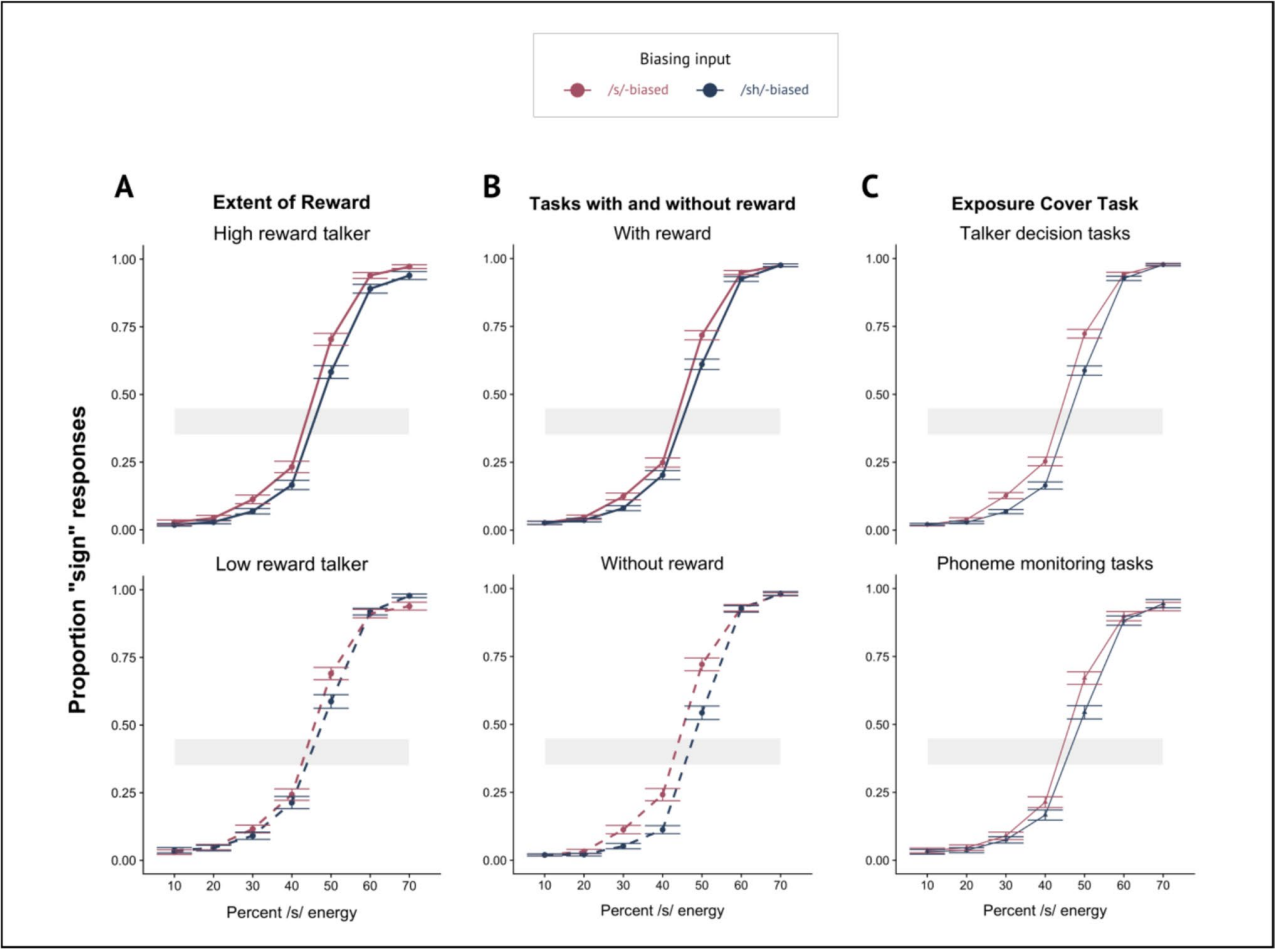
Plots of the pooled results for three post hoc analyses summarizing over phonetic categorization data from multiple experiments reported here. For all, the *x*-axis depicts the acoustic energy as the proportion of /s/ energy, proceeding from more /ʃ/-like responses on the left to more /s/-like responses on the right-hand side. The *y*-axis plots the mean of participant /s/ responses. Error bars depict the standard error of the mean. Bias direction is indicated by the color of the line, with red indicating the /s/-biased talker and the blue indicating the /ʃ/-biased talker. **A** Post hoc analysis comparing all experiments with a high- and low-reward talker to assess if the magnitude of the bias effect is different for the value of the talker. **B** Post hoc analysis comparing experiments with and without reward components to test if tasks with reward makes the bias effect smaller. **C** Post hoc analysis comparing magnitude of the bias effect after either the talker-decision cover task or the phoneme-monitoring cover task during exposure to examine any task-related differences in phonetic recalibration. (Color figure online)

Across multiple experiments with different implementations of reward, the value of each talker did not impact phonetic recalibration effects. This was unexpected and motivated several post hoc analyses to better understand the relationship between value-driven selective attention and talker-specific learning across all five experiments (see Table 4, Fig. 5, Fig. 6). We first examine, within the three experiments that included reward, if there was a significant difference in the size of the bias effect between the high-reward talker and the low-reward talker, regardless of the type of cover task used during exposure (total *n* = 320; see Fig. 6A; Table 4A). When pooling the data from Experiment 1A, 2B, and 3, we report a significant main effect of both Step (β = 5.01, *p* < 0.001) and Bias (β = 0.53, *p* < 0.001), but no significant interaction between Bias and Rewarded Talker (β = 0.27, *p* = 0.486). Even when maximizing the power to detect an effect of Rewarded Talker on the Bias effect, there was no evidence that the value of the talker during exposure translated to changes in the magnitude of the phonetic recalibration effect at test.

**Table 4:** Post hoc analyses

**A.** Reward magnitude comparison (all experiments with reward: Experiment 1A, 2B, and 3)					
SH_resp ~ Step × Bias × Rewarded Talker + (Step × (Bias + Rewarded Talker | Subject)					
**Fixed effects**	**Estimate**	**Std. error**	***Z* value**	***p* value**	**Significance**
Step	5.01	0.11	43.68	< 0.001	***
Bias	0.53	0.12	4.50	< 0.001	***
Rewarded Talker	0.080	0.12	0.68	0.50	
Step × Bias	0.04	0.12	0.36	0.72	
Step × Rewarded Talker	0.01	0.12	0.1	0.92	
Bias × Rewarded Talker	0.27	0.38	0.70	0.49	
Step × Bias × Rewarded Talker	− 0.29	0.46	− 0.63	0.53	
**B.** Experiments with reward (1A, 2B, 3) and experiments without reward (1B, 2A)					
SH_resp ~ Step × Bias × Reward + (Step × (Bias + Rewarded | Subject)					
**Fixed Effects**	**Estimate**	**Std. error**	***Z* value**	***p* value**	**Significance**
Step	5.12	0.10	50.66	< 0.001	***
Bias	0.84	0.11	7.66	< 0.001	***
Reward	− 0.33	0.16	− 2.01	0.04	*
Step × Bias	0.29	0.12	2.37	0.2	*
Step × Reward	− 0.24	0.20	− 1.21	0.23	
Bias × Reward	− 0.58	0.22	− 2.67	0.01	**
Step × Bias × Reward	− 0.42	0.24	− 1.72	0.085	
**C.** Experiments with the talker decision exposure task (1A, 1B, data reproduced from Luthra et al., 2021a, 2021b) and the phoneme monitoring exposure task (Experiments 2A, 2B, 3)					
SH_resp ~ Step × Bias × Exposure Task + (Step × (Bias + Rewarded Talker | Subject)					
**Fixed effects**	**Estimate**	**Std. error**	***Z* value**	***p* value**	**Significance**
Step	5.07	0.10	52.02	< 0.001	***
Bias	0.73	0.10	7.17	< 0.001	***
Exposure Task	0.30	0.16	1.91	0.0559	
Step × Bias	0.23	0.11	2.07	0.0385	*
Step × Exposure Task	− 0.04	0.20	− 0.18	0.85	
Bias × Exposure Task	− 0.18	− 0.20	− 0.91	0.36	
Step × Bias × Exposure Task	0.003	0.22	0.02	0.99

As part of Question 1, in a comparison between an experiment with reward (Experiment 1A) and one without (Experiment 2B), there was a marginally significant interaction suggesting that including any reward at all may have made the bias effect *smaller*. This outcome, paired with the trend from Experiment 2 in which, when the probabilistic reward schedule was used in conjunction with the phoneme-monitoring cover task, participants failed to engage in talker-specific phonetic recalibration writ large, motivated us to assess whether including reward in a lexically guided perceptual learning paradigm actually attenuates the size of the bias effect. We collapsed across tasks to compare the magnitude of the bias effect across all experiments without reward (Experiments 1B, 2A, data from Luthra et al., 2021a, 2021b) and those with reward (Experiments 1A, 2B, and 3), plotted in Fig. 6B and model output in Table 4B. There was a significant interaction between Bias and Reward (β =  − 0.58, *p* < 0.01), but participants indeed showed a *smaller* phonetic recalibration effect in experiments with reward than those without. We offer several interpretations of this finding in the discussion.

Model syntax on Line 2. Significance is indicated by asterisk number adhering to classic convention: *p* < 0.001 (***), *p* < 0.01 (**), *p* < 0.05 (*), *p* > 0.05 ( +), marginal significance (.)

Finally, as we report results from two different exposure cover tasks (gender decision and phoneme monitoring), we test whether the task used during the exposure phase affects the magnitude of the bias effect during phonetic categorization by comparing all experiments using the talker-decision task (Experiment 1A, 1B, and data from Luthra et al., 2021a, 2021b) versus those using the phoneme-monitoring task (Experiments 2A, 2B, and 3), plotted in Fig. 6C and model output in Table 4C. There was a lack of a significant interaction between Bias and the type of exposure task, indicating that the type of task did not modulate the degree of phonetic recalibration (β =  − 0.18, *p* = 0.36).

## Discussion

In the current multi-talker lexically guided perceptual learning study, we systematically tested whether manipulating the extrinsic value of each talker during exposure results in corresponding changes in the magnitude of the phonetic recalibration effect at test. We predicted that listeners would preferentially learn the phonetic details of a high-value talker compared with a low-value talker. However, using both probabilistic and deterministic reward schedules across multiple exposure tasks, we report that, on balance, extrinsic reward did not differentially change the size of the learning effect. A post hoc analysis further indicates that including extrinsic reward at all in a lexically guided perceptual learning paradigm may actually attenuate the bias effect; a finding that is surprising when considering work showing that reward generally improves perceptual learning (Bennett et al., 2019; Bonner & Sprinkle, 2002; Vartak et al., 2017; P. Zhang et al., 2018). Strikingly, Samuel (2016) found that if a secondary task requires listeners to engage cognitive resources within approximately 1 s after a to-be-recalibrated phoneme, perceptual learning does not occur; one possibility is that processing reward may similarly consume cognitive resources, interfering with recalibration. In our data, we found that the presence of a reward generally reduced the extent of perceptual learning, except when the reward occurred before the speech signal (Experiment 3); in that case, reward led to a relatively large learning effect, but only at one end of the continuum. Future work will be needed to more precisely characterize if the time course of reward influences the extent of perceptual learning.

Despite the unexpected pattern in the primary manipulation, we also successfully developed a novel cover task that results in talker-specific phonetic recalibration for multiple talkers—phoneme monitoring—that draws listener attention to the phonetic level of the speech stimuli without sacrificing learning (e.g., McAuliffe & Babel, 2016). Given the overall robustness of lexically guided perceptual learning reported here, the present data raise intriguing questions as to why perceptual learning for speech is seemingly imperturbable to reward, which might a priori be expected to shape how listeners attend to the speech signal.

At least two broad classes of interpretations emerge to explain the robustness of the learning effect observed here—and specifically, one that did not differ in size based on reward. One possibility is that perceptual adaptation in the speech domain, and its mechanisms, operate independently of reward. The other is that reward and the ways it interacts with attention do influence adaptation, but not in ways the manipulations implemented here tapped in to. The current findings cannot adjudicate between these explanations but inform each and point fruitfully towards future investigations.

Concerning the first possibility of the independence of adaptation and reward, Samuel (2016) posited that adjustments in sound-meaning correspondences are a natural consequence of lexical access. Under this viewpoint, while attention is certainly necessary as a means to facilitate word recognition, no additional resources (attentional or otherwise) need to be allocated for further adaptive processes. This captures our results, as all of the experiments presented here were designed to leave lexical access intact for both talkers. This account also provides a parsimonious explanation for other investigations which do not find learning to be affected by higher-level conscious value judgements (Babel, Senior, et al., 2019).

Not all findings, however, can be well situated under an account wherein adaptation is an inherent consequence of resolving lexical ambiguity. For example, a number of studies have found that when atypical pronunciations of words are dubbed onto videos of a talker holding a pen in their mouth, lexical endorsement is retained but lexically guided perceptual learning is blocked (Kraljic & Samuel, 2011; Kraljic et al., 2008; Liu & Jaeger, 2018). These results lend evidence that high-level knowledge—here related to the likelihood that the talker will continue to speak atypically once the pen is removed—can affect talker-specific learning. Our findings do not necessarily conflict with a system for adaptation which takes into account the potential value of adjustments before making them; however, the implementation of value in the current study may have not allowed us to observe potential influences of reward on phonetic recalibration.

For instance, in all experiments with a reward manipulation, participants received rewards for both the high- and low-reward talkers, leaving no condition in which a talker was not rewarded at all. This choice was deliberate as there are no “zero value” talkers in communicative contexts. However, it may be that the *difference* in the magnitude of the reward between high and low was not large enough to drive top-down changes in selective attention to modulate the degree of talker-specific recalibration. Studies that have used differential rewards during perceptual tasks have used 5:1 (Anderson et al., 2011; Anderson, 2016c; MacLean et al., 2016) and 10:1 (Mine & Saiki, 2015) ratios between high and low rewards and have found effects. It is possible that the reward difference of 3:1 between the high- and low-reward talkers in the current study was simply too small to reliably lead to corresponding changes in the size of the phonetic recalibration effect.

In Experiments 1A and 2B, the reward difference was induced by the probabilistic reward schedule such that one talker was rewarded 3 × as often as the other. While other experiments leveraging value-driven attentional capture have used probabilistic schedules (e.g., 4:1 in Mine et al., 2021), those schedules have simultaneously been convolved with reward magnitude, leaving open as to whether changes in selective attention during perception are more sensitive to variation in reward magnitude or reward probability. In Experiment 3, we decoupled reward magnitude and reward probability by cueing participants on every trial as to the value of the talker using a 3:1 ratio between high and low reward. The lack of an interaction between reward magnitude and the size of the bias effect suggests that, at least in this instantiation, that lexically guided perceptual learning may not be sensitive to reward magnitude in directing selective attention. Future iterations of this work may find success in driving value-based changes in phonetic recalibration if one talker is punished while the other is rewarded, though there is work showing that both rewards and punishments can bias selective attention during perceptual tasks (Le Pelley et al., 2019; Wang et al., 2013), leaving open as to whether or not listeners can be pushed to solely attend to input from one talker and disregard input from another.

Despite the issue of reward magnitude in driving perceptual learning, there is support for the idea that adjustments in sound–meaning correspondences may be sensitive to perceived talker value. In research of disordered speech, for example, exposure to educational information has been found to improve listeners’ attitudes towards people with dysarthria, but with no effect on transcription accuracy (Fletcher et al., 2023). While no opportunity to adapt to a dysarthric talker’s voice was provided after education, this finding shows that there may be a relationship between subjective evaluation of a talker and motivation to adapt to ambiguous speech. Additionally, studies of phonetic imitation and linguistic accommodation, which can be viewed as the speech production correlates of lexically guided perceptual learning, show differences in the degree of phonetic convergence—the degree to which talkers begin to sound like each other—depending on the social relationships between talkers. Specifically, asymmetries in power dynamics between talkers (e.g., Pardo et al., 2010), as well as the degree to which participants express an affinity for versus prejudice against their interlocutor (Adank et al., 2013; Babel, 2010, 2012) can affect the degree to which talkers will converge during conversation. This set of findings shows that the perceptual-to-articulatory loop responsible for context-dependent alterations in production is sensitive to social dynamics. One might suppose that social factors might more powerfully drive the speech production system, where the way a talker speaks reveals many details about her including social class, regional background, age, education, and so on. In contrast, perception may be less sensitive to these social pressures, as the outputs of perception are “invisible” to others.

The current collection of findings suggests a system where, at least in perception, listeners adapt whenever conditions do not actively suggest it would be detrimental to do so. Additionally, previous work suggests that this adaptation may already be maximal, as boosting lexical support through simultaneous lexical and semantic disambiguation does not increase the extent of learning (Luthra et al., 2021a, 2021b). Boosting the likelihood of reward for a particular talker may indeed increase a listener’s attention to and/or perceived evaluation of them, but these differences may not be visible against controls where learning effects are already at ceiling. This possibility positions the current work in good company, as many previous studies in lexically guided perceptual learning have found effects to be quite robust; even weathering manipulations explicitly designed to attenuate them (Baart & Vroomen, 2010; Babel, McAuliffe, et al., 2019; Samuel, 2016; X. Zhang & Samuel, 2014). The relative unflappability of the effect may be tied to the strength of lexical access as a prioritized and productive system for language in its own right, independent of adaptation (e.g., Cutler et al., 1987; Signoret et al., 2011). The possibility remains, therefore, that manipulations of attention and reward which do not affect lexically guided perceptual learning could still affect perceptual learning guided by alternative cues (e.g., semantics; Jesse, 2021). Evaluating and testing this possibility is left to future work. It does, however, further complicate exploration of how domain-general elements such as attention, motivation, reward, and executive function interact with perceptual learning, and other language tasks.

We would also like to acknowledge that, in principle, the learning effects observed here might be driven by multiple mechanisms. In the vast majority of lexically guided perceptual learning studies (e.g., Kraljic & Samuel, 2005; Norris et al., 2003), listeners are exposed to two kinds of critical stimuli: items containing ambiguous productions of one category (e.g., /s/) as well as items containing clear productions of a contrastive category (e.g., /∫/). The critical phonetic recalibration effect is assumed to be driven primarily by the ambiguous productions: Repeated exposure to ambiguous stimuli in lexically disambiguating contexts induces a shift in the perceptual boundary toward the critical category (e.g., the boundary moves toward /s/, such that there are more /s/ responses across the continuum). An open question, however, is the extent to which these learning effects might be driven by the unambiguous productions of the contrastive category. Previous work indicates that repeated exposure to a clear production of a speech sound (e.g., a clear /∫/) induces a shift away from that sound (e.g., the boundary shifts away from /∫/), a phenomenon referred to as selective adaptation; such adaptation effects may reflect fatigued responses by acoustic–phonetic feature detectors (Eimas & Corbit, 1973; Samuel, 1986; van der Zande et al., 2014; Vroomen et al., 2007). Critically, phonetic recalibration based on ambiguous stimuli and selective adaptation to clear stimuli from a contrastive category would both shift the phonetic category boundary in the same direction.

Given this, one might reasonably ask whether perceptual learning effects of this ilk might be driven by selective adaptation specifically—and furthermore, whether potential selective adaptation effects might be more pronounced in lexically guided perceptual learning studies like the current one, which do not include filler items (e.g., all items contain a fricative sound; Luthra et al., 2023, 2021a, 2021b). Previous studies provide some hints, with evidence that perceptual learning effects are comparable in size when clear productions of contrastive phonemes are or are not presented during exposure (Jesse, 2021) as well as when filler words are or are not presented during exposure (Luthra et al., 2021a, 2021b). However, to our knowledge, there has not been a formal investigation of how selective adaptation and phonetic recalibration contribute to perceptual learning, as well as whether their contributions might differ depending on whether exposure stimuli also include filler items. (The contributions of selective adaptation, for example, might be diluted when there are more filler items.) While one could conceivably attempt to isolate effects of phonetic recalibration by never including clear productions of contrastive phonemes, we would caution against doing so; with such a design, shifts in category boundaries might reflect learning of the distributional statistics of the exposure phase (e.g., that the talker never says the sound /∫/), such that effects at test might reflect a simple “probability matching” strategy (e.g., a decision-level adjustment rather than perceptual learning per se; Clarke-Davidson et al., 2008; Xie et al., 2017). Overall, disentangling the contributions of phonetic recalibration and selective adaptation is difficult, and we defer this question to future work.

As we consider the potential effects of reward on speech perception, it is useful to consider the extent to which successful speech perception may in and of itself be rewarding. In the majority of perceptual tasks where effects of reward have been observed, stimuli have consisted of low-level sensory objects such as visual shapes and spoken letters (e.g., Anderson et al., 2011, 2012). In the case of speech perception, however, listeners can leverage a variety of supervisory signals including phonetic category knowledge, lexical knowledge, and contextual knowledge to guide interpretation of the signal (Kleinschmidt et al., 2015). It may be the case that successful speech perception generates intrinsic reward signals, which in turn drive adaptation and obscure the influence of any extrinsic reward manipulations. Consistent with this proposal, previous work has demonstrated that internally generated confidence signals can predict participant performance on perceptual learning tasks (Guggenmos et al., 2016), suggesting that intrinsic rewards may be sufficient to guide learning. This raises the possibility that effects of extrinsic reward may only emerge in cases where speech perception is made less inherently rewarding, perhaps by making comprehension more challenging. In doing so, we may be better able to elucidate the role of reward during speech perception.

## Concluding remarks

In summary, we showed, across a series of experiments, that listeners do not preferentially learn the phonetic details of a talker that is rewarded more often than another talker during lexically guided perceptual learning. Although it is intuitive that listeners may be biased toward a talker that holds more value to them, such as a talker they are likely to encounter again, the evidence reported here does not support that hypothesis. Despite the phonetic recalibration effect being impervious to value-driven manipulation, we did show that talker-specific learning is remarkably robust across multiple exposure tasks and reward implementations, providing further support that lexically guided perceptual learning may tap into a core mechanism at the heart of speech perception. These results open new avenues for investigation into how classically domain-general processes including attention, motivation, and reward learning may factor into perceptual learning of speech.

## Supplementary Information

Below is the link to the electronic supplementary material.

Supplementary file1 (DOCX 116 KB)

## Data Availability

The datasets, scripts, and audio stimuli relevant to the current research are available for download in a public OSF repository (https://osf.io/y52ge/).

## Footnotes

1 As mentioned in the Introduction, the reward manipulation employed here was implicit rather than explicit. Nevertheless, a subset of astute subjects were able to discern which talker was rewarded more often than the other. To assess reward awareness, we asked whether subjects were explicitly aware of the reward structure by the end of the task. Exploratory analyses, which subset the sample to only those subjects who were aware of which talker was rewarded more often, qualitatively align in all cases with the analyses reported here and can be found in the Supplementary Materials in Supplementary Table 1.

2 We originally collected data from only 64 participants for Experiment 1A. When we analyzed this data, we found a significant interaction between bias and rewarded talker, such that participants appeared to learn more for the high-reward talker than for the low-reward talker, which is exactly what we predicted. However, despite the robustness of lexically guided perceptual learning writ large (Drouin & Theodore, 2018; Eisner & McQueen, 2006; Leach & Samuel, 2007; Maye et al., 2008; McQueen et al., 2006), we elected to internally replicate Experiment 1A, twice, to determine if the original significant effects were real or spurious. Ultimately, as reported under the Question 1 Results section, we found that there was no interaction between bias and rewarded talker, suggesting that the original significant effect was likely a result of random noise and did not reflect a meaningful change in behavior. Our conclusions would be wholly different if we had stopped after the first sample for Experiment 1A. We likely would not have run Experiments 2A–3, resulting in an erroneous and impoverished understanding of the nature of reward, motivation, and attention during talker-specific phonetic recalibration. We include this as a note of caution to encourage other researchers to prioritize internal replications, especially when developing novel manipulations.

3 To note, this age range only includes participants collected from the Prolific sample. The 16 participants from the participant pool sample were all over 18 years old and were undergraduate students at the University of Connecticut, but we do not have additional age data from that group.

4 While the main effect of Bias did not reach significance in this sample and did in the parallel sample of Experiment 1A, post hoc analysis (available via the OSF repository associated with this manuscript) finds that the effect of bias does not meaningfully differ between experiments. We interpret this as a case where differences in significance do not imply significant differences (e.g., Gelman & Stern, 2006); for example, while the effect of bias straddles the *p* value of .05 between Experiments 1A and 2B, we do not believe that the task qualitatively affected the learning process to a meaningful degree. This argument is further supported by our post-hoc summary analyses (see Fig. 6 C).
