# Supplementary material for: Cents and shenshibility: The role of reward in talker-specific phonetic recalibration
Source: Atten Percept Psychophys. 2025 Apr 11;88(2):52. doi: 10.3758/s13414-025-03048-z (PMC12864238; doi:10.3758/s13414-025-03048-z)
Supplement: Supplementary file 1 — Supplementary file1 (DOCX 116 KB) [file 13414_2025_3048_MOESM1_ESM.docx]

**Supplementary Materials**

| **Supplementary Table 1** Phonetic categorization results for participants aware of which   talker was rewarded more than the other in Experiment 1A | | | | | |
| --- | --- | --- | --- | --- | --- |
| SH_resp ~ Step * Bias * Rewarded Talker + (Step * (Bias + Rewarded Talker \| Subject) | | | | | |
| **Fixed Effects** | **Estimate** | **Std. Error** | **Z value** | **p value** | **significance** |
| Step | 4.80 | 0.15 | 31.57 | < 0.001 | *** |
| Bias | 0.41 | 0.18 | 2.29 | < 0.05 | * |
| Rewarded Talker | 0.397 | 0.18 | 2.24 | < 0.05 | * |
| Step x Bias | -0.16 | 0.19 | -0.88 | 0.378 |  |
| Step x Rewarded Talker | 0.32 | 0.19 | 1.72 | 0.085 | . |
| Bias x Rewarded Talker | 0.12 | 0.51 | 0.24 | 0.81 |  |
| Step x Bias x Rewarded Talker | 0.19 | 0.61 | 0.31 | 0.75 |  |
| Model syntax on line 2. Significance is indicated by asterisk number adhering to classic convention (*p* < 0.001 (***), *p* < 0.01 (**), *p* < 0.05 (*), *p* > 0.05 (+), marginal significance (.) | | | | | |

| **Supplementary Table 2** Phonetic categorization results from Experiment 1B | | | | | |
| --- | --- | --- | --- | --- | --- |
| SH_resp ~ Step * Bias * Rewarded Talker + (Step * (Bias + Rewarded Talker \| Subject) | | | | | |
| **Fixed Effects** | **Estimate** | **Std. Error** | **Z value** | **p value** | **significance** |
| Step | 4.99 | 0.22 | 22.40 | < 0.001 | *** |
| Bias | 1.31 | 0.27 | 4.81 | < 0.001 | *** |
| Rewarded Talker | -0.36 | 0.27 | -1.33 | 0.18 |  |
| Step x Bias | 1.08 | 0.30 | 3.58 | < 0.001 | *** |
| Step x Rewarded Talker | 0.23 | 0.30 | 0.76 | 0.45 |  |
| Bias x Rewarded Talker | -1.11 | 0.76 | -1.46 | 0.14 |  |
| Step x Bias x Rewarded Talker | -0.39 | 0.89 | -0.44 | 0.66 |  |
| Model syntax on line 2. Significance is indicated by asterisk number adhering to classic convention (*p* < 0.001 (***), *p* < 0.01 (**), *p* < 0.05 (*), *p* > 0.05 (+), marginal significance (.) | | | | | |

As noted in the main text, the visual effect of bias appears to reverse between the high- and low- reward talker conditions of Experiment 3 (see Figure 4, reproduced below). However, as the effect is limited to one end of the continuum, the crucial Bias by Rewarded Talker interaction does not reach significance in our *a priori* specified maximal model structure (β = 0.68, p = 0.538); note that there is no a priori reason why we would expect a modulatory influence of reward at only one end of the continuum In other words, the third order interaction of Continuum Step by Bias by Rewarded Talker, which is significant in the maximal model (β = -2.59, *p* = 0.025), may be soaking up variance and hiding the potential impact of Rewarded Talker.


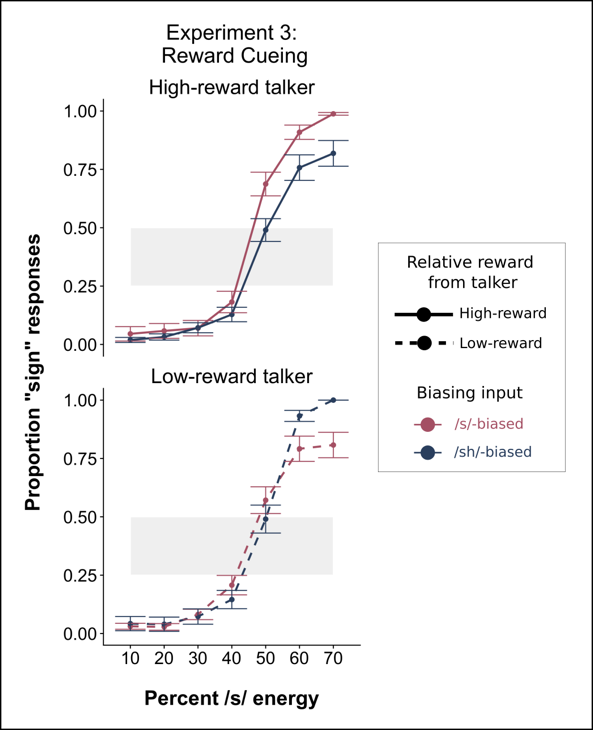


**Fig. 4.** (reproduced from main text)

Below we explore this possibility with two additional models and provide some discussion of potential implications. These analyses are of course exploratory and entirely post-hoc, and should be viewed as exciting pointers towards future work rather than strong evidence for conclusions to be drawn from the current data.

First, we constructed a model parallel to that employed in the main text, but removed the Continuum Step by Bias by Rewarded Talker interaction. Results of this model can be seen in Supplemental Table 2. In this model, the Bias by Rewarded Talker interaction increases in numerical magnitude (β = 1.21, up from 0.68), but does not reach significance (*p* = 0.266).

| **Supplementary Table 2** Phonetic categorization results from Experiment 3 with removed 3-way   interaction | | | | | |
| --- | --- | --- | --- | --- | --- |
| SH_resp ~ Step + Bias + Rewarded Talker + Step*Bias+ Step*Rewarded Talker + Bias*Rewarded Talker + (Step * (Bias + Rewarded Talker \| Subject) | | | | | |
| **Fixed Effects** | **Estimate** | **Std. Error** | **Z value** | **p value** | **significance** |
| Step | 4.89 | 0.29 | 16.92 | < 0.001 | *** |
| Bias | 0.66 | 0.26 | 2.52 | < 0.05 | * |
| Rewarded Talker | -0.07 | 0.26 | -0.27 | 0.79 |  |
| Step x Bias | 0.21 | 0.29 | 0.73 | 0.47 |  |
| Step x Rewarded Talker | -0.11 | 0.29 | -0.38 | 0.71 |  |
| Bias x Rewarded Talker | 1.21 | 1.09 | 1.11 | 0.27 |  |
| Model syntax on line 2. Significance is indicated by asterisk number adhering to classic convention (*p* < 0.001 (***), *p* < 0.01 (**), *p* < 0.05 (*), *p* > 0.05 (+), marginal significance (.) | | | | | |

Second, we consider a model where continuum step is removed entirely as a predictor. However, there are a number of theoretical reasons why this sort of model should be dispreferred. As listeners accommodate the span of tokens presented (e.g. Yamada & Tohkura, 1992), presented acoustics are an important consideration. Additionally, continuum step is the strongest individual predictor of “sh” responses in our data, and is usually stronger than the effect of learning observed in this paradigm. Unsurprisingly, therefore, model comparison operationalized via ANOVA suggests that removal results in significantly worse model fits (*p* = 0.030). Finally, there are strong reasons step should interact with learning, as both theoretical accounts (e.g. Kleinschmidt & Jaeger, 2015) and empirical results (Babel et al., 2019) assert learning to be strongest for tokens perceived as maximally ambiguous.

With all the disclaimers above noted, it is nevertheless our central interest whether there is any hint of evidence towards a relationship between learning and talker reward. Further, this sort of analysis may in principle be better-powered to test for the interaction of interest, though in the absence of an expected effect size, we defer formal power analyses to future work. We did therefore conduct a parallel analysis excluding continuum step as a predictor, and in this model (see Supplementary Table 3), the interaction of Bias and Rewarded Talker reached significance (β = 1.44, *p* = 0.021).

| **Supplementary Table 3** Phonetic categorization results from Experiment 3 with Step removed as a   predictor | | | | | |
| --- | --- | --- | --- | --- | --- |
| SH_resp ~ Bias*Rewarded Talker + (Bias + Rewarded Talker \| Subject) | | | | | |
| **Fixed Effects** | **Estimate** | **Std. Error** | **Z value** | **p value** | **significance** |
| Bias | 0.13 | 0.05 | 2.53 | < 0.05 | * |
| Rewarded Talker | -0.01 | 0.05 | -0.13 | 0.89 |  |
| Bias x Rewarded Talker | 1.44 | 0.62 | 2.32 | < 0.05 | * |
| Model syntax on line 2. Significance is indicated by asterisk number adhering to classic convention (*p* < 0.001 (***), *p* < 0.01 (**), *p* < 0.05 (*), *p* > 0.05 (+), marginal significance (.) | | | | | |

To the extent that the results of this final model indicate a modulating effect of reward on learning, at least two possibilities are forwarded. Experiment 3 deviated from the earlier manipulations both in the extent and the timing of presentation of reward. If the relationship relates to the extent of reward, then (as a reviewer helpfully pointed out), “if you give people really certain information that it may not be worth their while to even listen to half of the stimuli, maybe they can tune those out sometime”.

Alternately, if the relationship rather relates to timing of reward presentation, then perhaps this Experiment served as an unintentional dual task paradigm. Specifically, prior work has found auditory distraction, when presented at the right moment, to interrupt learning (Samuel, 2016; Jesse & Kaplan, 2019). Importantly, concurrent visual stimulus has never been shown to affect adaptation (see Hodges et al., under review), but the possibility nonetheless remains that the text presentation of Experiment 3 (immediately before stimulus presentation) served as such a blocker. We leave both possibilities to future work, designed and powered specifically to detect and extricate these factors. Overall, however, our work suggests that the influence of reward on phonetic adaptation is small, if present.

**References:**

Babel, M., McAuliffe, M., Norton, C., Senior, B., & Vaughn, C. (2019). The Goldilocks zone of perceptual learning. *Phonetica*, *76*(2-3), 179-200.

Hodges, E., Cummings, S., & Theodore, R. M. (2024, September 13). Lexically guided perceptual learning is robust to linguistically engaging distraction. <https://doi.org/10.31234/osf.io/q2mhs>

Jesse, A., & Kaplan, E. (2019). Attentional resources contribute to the perceptual learning of talker idiosyncrasies in audiovisual speech. *Attention, Perception, & Psychophysics*, *81*(4), 1006-1019.

Kleinschmidt, D. F., & Jaeger, T. F. (2015). Robust speech perception: recognize the familiar, generalize to the similar, and adapt to the novel. *Psychological review*, *122*(2), 148.

Samuel, A. G. (2016). Lexical representations are malleable for about one second: Evidence for the non-automaticity of perceptual recalibration. *Cognitive psychology*, *88*, 88-114

Yamada, R. A., & Tohkura, Y. (1992). Perception of American English/r/and/l/by native speakers of Japanese. *Speech perception, production, and linguistic structure*, 155-174.
